# Acoustic and Multimodal Deterrents to Limit Invasive Fish Passage

**DOI:** 10.1093/icb/icag137

**Published:** 2026-08-05

**Authors:** Allen F Mensinger

**Affiliations:** Department of Biology, University of Minnesota Duluth, Duluth, MN 55812, USA

## Abstract

Acoustic deterrents have been deployed with variable success to decrease fish interactions with anthropogenic hazards (i.e., power plant intakes and spillways) and prevent the spread of invasive species. Acoustic deterrents are nonphysical barriers that offer many advantages in that sound can travel a great distance, are relatively independent of light and weather conditions, do not interfere with navigation and are relatively inexpensive to implement. Assessing the efficacy of deterrents is challenging as there are few controlled or long-term studies and many reports fail to specify sound parameters (i.e., intensity, frequency) preventing independent assessment. It is imperative to factor fish sensory physiology when designing nonphysical deterrents as fish need to detect, localize, and move away from the stimulus. The auditory sensitivity of the target species must be identified to optimize the acoustic stimulus and the ambient soundscape mapped to ensure the sound intensity will be effective. Animals may habituate to an aversive stimulus and therefore multimodal deterrents may prove more effective than single-modal systems. This paper will provide an overview of acoustic deterrents, species targeted, and their efficacy. Special emphasis will be given to efforts to block the upstream migration of invasive bigheaded carp in the Mississippi River basin that currently threatens the Laurentian Great Lakes. Recent studies using a multimodal acoustic deterrent that conditions fish to associate sound with carbon dioxide will be described.

## Introduction

Deterrents have been deployed with intermittent success to decrease fish interactions with anthropogenic hazards and to prevent the spread of invasive species (Putland and Mensinger 2019a). The human desire to convert free-flowing waterways into sources of hydroelectric power, flood control, irrigation reservoirs, and improve navigation has resulted in transriver barriers that present anthropogenic challenges for fishes. Water flows into power plants for cooling or through hydroelectric dams to generate power often leads to deleterious consequences for fish caught in these currents. Oceans and rivers also provide dispersal highways for aquatic invasive species to quickly expand their ranges, however, large waterfalls historically provided formidable obstacles for upstream fish migration of both native and invasive species. Many of these waterways have evolved into vital transportation highways, and canals and/or navigation locks allow both ships and fish to bypass natural (i.e., waterfalls) or manmade (i.e., dams) barriers. The invasion of the sea lamprey (*Petromyzon marinus)* into the Laurentian Great Lakes through the Welland Canal and the subsequent decimation of the lake trout (*Salvelinus namaycush*) provides one of the most infamous examples of how altering waterways can produce ecological damage (Hubbs and Pope 1937).

Physical deterrents such as screens or barriers can reduce fish deaths at water intakes and/or block fish passage; however, they are prone to clogging, require constant maintenance, and may be circumvented during flooding. High head dams often have adjacent navigation lock chambers that allow both native and invasive fishes to bypass the barrier. Therefore, numerous attempts have been made to develop “nonphysical” deterrents such as light, sound, and/or odor to deter fish from water intakes or moving upstream through navigational locks. Unfortunately, the efficacy of these deterrents is difficult to assess as there have been few controlled studies or long-term monitoring. The success rate of acoustic deterrent varies substantially, ranging from failure to controlling behavior, to demonstrations that a few species might be stopped under very specific conditions. Compounding the variability is the absence of critical details such as frequency, range, sound pressure level, particle motion, or equipment specifications. The lack of quantitative data and peer-reviewed publications combined with proprietary stimuli and authors conflict of interest makes accurate third-party verification of the efficacy of deterrents difficult (Putland and Mensinger 2019a).

Further complicating the deterrent field is the considerable variation in how success is defined. For example, reducing fish kills due to impingement in water intakes by 20% may be considerable moderate success, however, deterrents that only stop 90% of invasive species will not prevent upstream spread and reproduction. Also, the fish’s life history and the frequency with which it challenges the deterrents need to be factored. Anadromous fish (i.e., salmon) or catadromous fish (i.e., eels) can face a gauntlet of anthropogenic hazards as they move downstream, however, prevailing water currents may expose them to each hazard only once. In comparison, resident fish populations near water intakes or invasive fish migrating upstream for reproductive or nutritional advantages, may need to be repeatedly repelled and therefore could eventually habituate to the stimulus. Thus, a single system may need to be dynamic and continually alter the stimulus (i.e., sound frequency) or multimodal deterrents that employ two aversive stimuli could achieve greater success.

A recent paper reviewed 286 studies on 111 species and their interactions with acoustic deterrents. Approximately 11% of the studies reported < 50% deterrence, 26% reported > 50% deterrence, and the remaining studies not quantifying efficacy. Less than 7% of the studies examined habituation with results evenly split between habitation and no habituation. (Putland and Mensinger 2019a) Table 1. These results reflect that simply projecting sound at fish may not affect behavior and that future deployments need to consider fish physiology and the prevailing soundscape with long-term monitoring system to continue to assess efficacy.

**Table 1:** Summary on use of acoustic deterrents for different fish orders.

Order (studies)	Species tested	Auditory sensitivity of example species (Hz)	Deterrent frequency range (Hz to kHz)	Deterrent SPL range (dB re 1µPa) kHz	Efficacy (%)		Habituation tested	
Clupeiformes (66)	13	200–4000, 10,000 to 100,000 *(Brevoortia patronus)* ^ab^	20–198	72–202	<50%	5.9	No	2.9
					>50%	27.9	Yes	1.5
					NR	66.2	NR	95.6
Cypriniformes (49)	23	100– 7000 (*Hypophthalmichthys molitrix*)^c,d^	16–150	120–172	<50%	12.7	No	9.1
					>50%	49.1	Yes	7.3
					NR	38.2	NR	83.6
Perciformes (52)	25	10–500 (*Perca fluviatilis*)^e^	20–145	132–172	<50%	7.7	No	0.0
					>50%	19.2	Yes	0.0
					NR	73.1	NR	100.0
Salmoniformes (57)	17	10 –1000 (*Salmo salar*)^f,g^	5–6.4	50–194	<50%	5.3	No	5.3
					>50%	22.8	Yes	5.3
					NR	71.9	NR	89.5
Anguilliformes (n9)	1	10–300h (*Anguilla anguilla*)^h^	10–128	155–172	<50%	22.2	No	0.0
					>50%	22.2	Yes	11.1
					NR	55.6	NR	89.9
Gadiformes (8)	4	50–300^ij^*(Gadus morhua)*	30–128	155–214	<50%	12.5	No	0.0
					>50%	12.5	Yes	0.0
					NR	75.0	NR	100.0
Pleuronectiformes (7)	5	10–300k *(Pleuronectes platessa)*	20–128	155–172	<50%	71.4	No	0.0
					>50%	0.0	Yes	0.0
					NR	28.6	NR	100.0
Other (38)	23	10–5000^lmn^ various	20–128	135–172	<50%	18.4	No	0.0
					>50%	15.8	Yes	0.0
					NR	65.8	NR	100.0

Modified from Putland and Mensinger (2019a). Willis et al. (2002).

ab Mann et al. (1997) and Mann et al. (2001).

cd Lovell et al. (2006) and Vetter et al. (2018).

e Karlsen (1992).

fg Vanderwalker (1967) and Hawkins and Johnstone (1978).

h Jerkø et al. (1989).

ij Chapman and Hawkins (1973) and Chapman and Sand (1974).

lmn Kelly and Nelson (1975), Casper and Mann (2006), and Hawkins (1981).

This review will focus on acoustic deterrents in aquatic habitats, discuss how fish auditory sensitivity must be factored in deterrent design, and examine the efficacy of acoustic deterrents over a wide range of native and invasive species, before detailing recent experiments using a multimodal deterrent against invasive bigheaded carp. Limitations, environmental implications and future directions for acoustic deterrents will be discussed.

## Fish sensory physiology and deterrents

For nonphysical deterrents to be effective, fish must be able to detect, locate and be “guided” away from the deterrent with a need for long-term monitoring systems to evaluate success. Visual, tactile olfactory, or auditory cues could be deployed alone or in combination to guide or repel fish away from targets. Sensory gradients may be the most effective as further movement into the gradient should elicit increasing negative stimulus and encourage fish to swim away from the source. However, sensory systems can habituate to a single stimulus and potentially ignore repeated applications and therefore the deterrent should have dynamic flexibility (i.e., changing sound frequencies/intensities) or be paired with a second stimulus. The intensity level needs to be sufficient to evoke continual repulsion while preventing chronic damage to sensory organs.

It is imperative to factor fish sensory physiology into the design of nonphysical deterrents. For example, several studies have implemented strobe lighting with acoustic deterrents to repel fish without factoring the flicker fusion frequency of the retina to determine whether it perceives the strobes as intermittent flashes or continuous light. Additionally, white light is often used, instead of matching the wavelength with the fish’s visual sensitivity which would increase detection sensitivity and perception distance (Ruebush et al. 2012; Kim and Mandrak 2017). For acoustic deterrents, it is imperative to understand the auditory sensitivity of the target species as several studies that reported failure used sound frequencies outside the fish’s hearing range (Putland and Mensinger 2019a; Gibson and Myers 2002).

## Sound and fish auditory systems

Understanding sound properties is imperative when considering acoustic deterrents. Sound is a waveform that travels through a medium accompanied by a transfer of energy from point to point (Urick 1983). The waves consist of alternating pressure deviations, which cause localized regions of compression and rarefaction, called *sound pressure*. Individual particles, however, do not travel with the wave, but vibrate back and forth, causing *particle motion*. Underwater sound is further divided into near-field, which is within approximately two wavelengths of the sound source, and the far-field, which is outside the near-field. Close to the source, sound waves behave in a complex fashion because sound pressure is not in phase with the particle motion component (Higgs and Radford 2016), whereas outside the near-field, particle motion is in phase with sound pressure, and the sound intensity decreases by approximately 6 dB for each doubling of distance from the source (Urick 1983; Putland and Mensinger 2019a).

The otolith organs in the fish inner ear are the primary auditory organs and detect particle motion directly via an inertial response of the otoliths to motion (Popper and Fay 1993). All teleosts generally detect particle motion less than 1 kHz while ostariophysans with Weberian ossicles or other species with ancillary hearing structures can detect sound pressure and extend frequency detection up to 5 kHz. Sound pressure sensitivity depends on the presence of air-filled cavities in the body that can serve as pressure-to-motion transformers (Braun and Grande 2008; Deng et al. 2011; Parmentier and Fine 2016). Accordingly, auditory sensitivity varies considerably among species in relation to the presence and morphology of the swim bladder, its proximity to the inner ear, or the presence of specialized anatomical structures from the swim bladder to the inner ear (i.e., Weberian ossicles) (Fay and Popper 1978; Hawkins and Myrberg 1983). Thus, auditory sensitivity in teleosts is a continuum that can range from infrasound (< 20 Hz) to approximately 5 kHz with a few specialized species (Clupeiformes) able to detect ultrasound (> 20 kHz) (Mann et al. 2001, 2005; Popper et al. 2022). Ostariophysian such as Cyprinids (carps and minnows) have a series of bones called Weberian ossicles that acoustically couple the vibrations from the swim bladder to the sacculus of the inner ear. The bony ossicles amplify sound waves and allow cyprinids to perceive a greater range of auditory stimuli than other freshwater fishes (Popper and Fay 1993). For example, common (*Cyprinus carpio)*, bighead (*Hypophthalmichtys nobilis*) and silver carp (*H. molitrix)*, detect sound up to 5 kHz (Vetter et al. 2018). Differential frequency sensitivity provides the opportunity to acoustically target fishes with higher frequency sensitivity without impacting species that cannot detect sound pressure.

## Acoustic deterrents

Acoustic deterrents primarily consist of underwater speakers and/or bubble curtains, Bubble curtains are produced by injecting compressed air through a perforated tube on the bottom of the waterway and as the bubbles rise, air expands and creates pressure waves that generate popping sounds (Leighton and Walton 1987). Fish may be deterred by sound and/or tactile interaction with the bubble curtain alone, however, these deterrents are short range with sound pressure decreasing approximately 95% within 1 m of the source (Turnpenny and O’Keeffe 2005). Combining underwater speakers and bubble curtains create ensonified bubble curtains with sound trapped between the two bubble sheets in the water column. However, the efficacy of bubble currents can be degraded by water currents, vessel traffic, or fish swimming through gaps in the bubbles. In comparison, acoustic deterrents with underwater speakers produce an omnidirectional field of sound and can have a greater range than bubble currents due to greater sound intensity at the source and allow a sound gradient to establish a considerable distance from the speakers.

Acoustic deterrents are limited to small areas or relatively narrow passageways as it would be difficult to ensonify an entire river. Navigational lock chambers that allow ship transit provide an excellent location for acoustic deterrents. As high head dams block upstream passage, the lock chambers often provide the only pathway upstream during normal river conditions. The locks act to raise or lower vessels to the water levels on either side of the dam. The lock chamber and approach channel are well suited to confine and amplify the sound and establish a sound gradient and the acoustic deterrents should not pose a navigational hazard. The downstream lock gates open in advance of incoming shipping, close after the vessel opened, the lock, water levels are raised, and the upstream gate is opened allowing the ship to move upstream. Acoustic deterrents only need to be operational when the downstream gate is opened.

## Native fish

Many initial acoustic deterrents targeted native fishes to reduce mortality when interacting with anthropogenic hazards. Clupeids (herring and shad) can be impinged against water intake screens and as several species have ultrasonic auditory sensitivity (Mann et al. 2001, 2005), they could be targeted specifically without impacting other fishes. Ultrasound 9122 to –128 kHz, SPL 155–167 dB re 1 µPa), deterred blueback herring (*Alosa aestivalis*) from a hydroelectric power station in Annapolis, Nova Scotia (49% deterrence) (Gibson and Myers 2002) while alewives (*A. pseudoharengus*) impingement was reduced 87–97% (110–128 kHz, SPL 190 dB re 1 µPa) on a water intake in Lake Ontario, Canada (Ross et al. 1995).

Anadromous and catadromous fishes encounter a wide variety of anthropogenic challenges during freshwater migration. Their commercial and ecological importance has led to considerable investment in reducing mortality with fish ladders provided to circumvent obstructions on upstream passage. However, downstream migration exposes fish to water intakes, dams, spillways, and turbines. Non ostariophysians salmonids behavioral responses to sound have been mixed with steelhead (*O. mykiss*) and chinook (*O. tshawytscha)* salmon failing to respond to 10–500 Hz vibrations (max 160 dB re 0.0002 dynes/cm^2^) in the field (Vanderwalker 1967), however, a motion generator (10 and 150 Hz) repelled Atlantic salmon in a tank and a small diversion channel (Knudsen et al. 1992, 1994). A ~250 m transducer array (5–600 Hz, no SPL reported) diverted 50 to 80% of downstream migrating juvenile chinook salmon away from a slough, however, the efficacy decreased dramatically to 8–15% during flooding (Perry et al. 2012) indicating river conditions can influence the efficacy of acoustic deterrents. Catadromous European eels were deterred (up to 57%) by ~12 infrasound Hz stimulus (PAL ~ 0.01 m s^−2^ at 11.8 Hz at 3 m) (Sand et al. 2000) and a broadband (60–1000 Hz) pulsed sound guided European eel away from a water intake (Deleau et al. 2020).

Cyprinid’s increased auditory sensitivity has made them a target for acoustic modification with approximately half of the studies indicating at least 50% efficacy (Putland and Mensinger 2019a). Roach (*Rutilus rutilus*), common bleak (*Alburnus alburnus*), common bream (*Abramis brama*), common dace (*Leuciscus leuciscus*) and chub (*L. cephalus*) were deterred (68–88%) from a cooling water inlet in the UK (frequency and SPL not reported) (Wood et al. 1994) Field trials in the River Schelde estuary, Belgium showed 40.1% reduction of white bream (*A. bjoerkna*) (Maes et al. 2004) although, this was less than the 60% deterrence reported for noncyprinid species (i.e., perch, herring and sprat). In Lake Borrevann, Norway, 16 Hz infrasound resulted in a 80% reduction of individual fishes at 10 m away from the source in rudd (*Scardinius erythrophthalamus*), roach and common bleak, however, the study was limited to a single night, and sound intensity was not reported (Sonny et al. 2006). A laboratory study in Portugal showed strong repulsion to a sine sweep (up to 2000 Hz) but little reaction to intermittent 140 Hz tone (140 dB re 1 µPa) for northern straight mouth nase (*Pseudochondrostoma duriese*) and Iberian barbel (*Luciobarbus bocagei*) suggesting signal type for deterrence must be considered. However, the sweep signal varied greatly in amplitude across the frequency range tested due to nonlinearity of the speaker, preventing an accurate sound intensity from being determined (Jesus et al. 2019).

## Invasive fishes

Acoustic deterrents have also been proposed against invasive fishes. The Niagara Escarpment previously prevented sea lamprey migration (*P. marinus*) past Niagara Falls, however, the Welland canal allowed the sea lamprey to bypass the falls in the early 20th century and devastate the fisheries in the upper Great Lakes (Smith and Tibbles 1980). Current sea lamprey controls apply lampricides in streams, however, these involve extensive effort and expense and are detrimental to native lampreys. It has been hypothesized that sound could either attract lampreys to traps or deter upstream movement (Bravener and McLaughlin 2013; Heath et al. 2021). Despite its ancient lineage, it was only recently demonstrated that lamprey can respond to sound with low-frequency (70 to 90 Hz) sounds leading to increased activity while high-intensity sounds of similar frequencies (up to 160 dB re 1 µPa) can repulse lamprey in streams (Mickle et al. 2019; Heath et al. 2021, 2025).

Many species of carp are invasive to North America and include common (*Cyprinus carpio*), bighead (*Hypophthalmichtys nobilis*) silver (*H. molitrix)*, grass (*Ctenopharyngodon idella*) and black (Mylopharyngodon piceus) carp that detect sound up to 5 kHz (Vetter et al. 2018; Nissen and Mensinger 2023). These provide the opportunity to acoustically target these fishes with higher frequencies without impacting native non ostariophysans that respond to lower frequencies.

The common carp was introduced into the Midwest in the late 1800s as a food fish and its spread was accelerated by the US Commission of Fish and Wildlife stocking the fish throughout the continental United States (Balon 1995). It quickly degraded ecosystems by disturbing sediments and uprooting plants. Given its widespread distribution, complete eradication of the species is beyond current technology, however, deterrents could block access to spawning areas to prevent reestablishment after local extirpation (Bajer et al. 2022).

Acoustic deterrents can impede common carp movement, however, the results have been mixed. Bubble curtains (200 Hz, 130 dB re1 µPa), reduced common carp passage in an indoor channel by 75–85% (Zielinski et al. 2014) and yielding 59% downstream deterrence but only 11% upstream blockage and in Kohlman Creek, Maplewood, Minnesota, USA (Zielinski and Sorensen 2015). However, an acoustic deterrent (200–150 Hz) with a sound-pressure level of 158 dB re 1 µPa had little effect on common carp in fishway in Ontario, Canada (Bzonek et al. 2021b). During flume experiments, a bubble curtain showed 47% deterrence, that was increased to 65% with the addition of 1750 or 4000 Hz sound, (SPL ~ 130 dB), however, both curtains were less effective during the day, suggesting that vision plays an important role at mediating carp reactions (Martin et al. 2021). Speakers were mounted on the downstream gate of the lock chamber of Lock and Dam 8 on the Mississippi River and boat motor sounds (maximum SPL_RMS_ of 139 dB re 1 µPa at 654 Hz) yielded weak avoidance response from common carp undertaking upstream movements (Riesgraf et al. 2022). Stroboscopic and auditory stimuli in a boating slip (SPL 175 dB re 1 µPa) against common carp showed weaker responses compared to other studies (Bzonek et al. 2021a) presumably due to high SPL and lack of acoustic refuge. Sound sweeps ranging from 200 to –1500 Hz band sweep, (SPL–142 (dB) re 1 µPa) detained carp from leaving areas increased concentrations of CO_2_ (Bzonek et al. 2022).

Silver and bighead carp, collectively known as bigheaded carp were introduced to the southeastern United States for algae control but escaped into the Mississippi drainage where they have expanded both up and downstream and currently threaten the Laurentian Great Lakes (Kolar et al. 2007; Sass et al. 2010). Their filter feeding competes with native planktivores and disrupts the lower tiers of the food web for game fish. Carp have negatively impacted native paddlefish (*Polyodon spathula*) (Schrank et al. 2003), gizzard shad (*Dorosoma cepedianum*) (Sampson et al. 2009), and bigmouth buffalo (*Ictiobus cyprinellus*) (Irons et al. 2007) due to their fast growth, prolific spawning, and ability to outcompete native fish for food and space. They remain mostly confined to river systems and considerable resources have been invested to capture, deter, or eliminate these species. However, the fishes are adept at avoiding nets and the public’s disinterest in consumption makes commercial fishing a limited option. Some success has been found using a unified method which involves a fleet of small craft, multiple nets and sound generation to herd/drive fish into a collection area. While this may temporarily depress the population, the effort does not guarantee complete removal of the fish from water body and is labor intensive (Chapman 2020).

Acoustic deterrents against bigheaded carp were first tested in 2005 when an ensonified bubble curtain in a shallow concrete channel modified carp movement (Taylor et al. 2005). Unfortunately, a subsequent BAFF field deployment (500 to 2000 Hz, no SPL reported) in Quiver Creek, Illinois was unable to determine if fish challenged the barrier (Ruebush et al. 2012). Silver carp jump in the presence of motorized watercraft and it was hypothesized that sound alone could influence their behavior (Vetter et al. 2017a). Playbacks of boat motor sound (60–10,000 Hz, SPL of ~150 dB) in outdoor ponds consistently produced negative phonotaxis in bigheaded carp and prevented up to 94% of the carp from passing through a narrow 1 m channel (Vetter et al. 2015, 2017b; Murchy et al. 2017). Follow up studies showed there was a minimum effect on native fishes including: bigmouth buffalo (*I. cyprinellus*), channel catfish (*Ictalurus punctatus*), fathead minnow (*Pimephales promelas*), American eel (*Anguilla rostrata*), gizzard shad (*Dorosoma cepedianum*), hybrid striped bass (*M. saxatilis x M. chrysops*), lake sturgeon (*Acipenser fulvescens*), and paddlefish (*Polyodon spathula*) (Murchy et al. 2022). In a study in outdoor earthen ponds, synthesized chirp saw or squares influenced silver carp behavior more than outboard motor sounds. However, silver carp did not move away from the sound source, which was attributed to the physical scale of these ponds and relatively high sound pressure levels (170.0 dB re 1 µPa) (Faulkner et al. 2025).

These initial studies have lead to the field deployment of two large-scale acoustic deterrents. A BioAcoustic Fish Fence (BAFF) is a multimodal deterrent using an air-bubble curtain, light, and sound, and was deployed in 2019 at the downstream lock approach at Barkley Lock and Dam on the Cumberland River near Grand Rivers, Kentucky. The deterrent saw an approximate 50% reduction in the number of silver carp completing upstream passage through Barkley Lock when the deterrent was in operation (Fritts et al. 2023), however, sound parameters were not reported. An underwater acoustic deterrent system (UADS) was installed at Lock 19 on the Mississippi River in 2021. Sixteen underwater speakers were recessed in the downstream water discharge and operated on 80 hours on/off cycle with silver carp twice as likely to pass over the UADS and into the lock when it was off than with engineered sounds (Brey et al. 2023). The engineered sound parameters are available in a pending patent (Woodley, CM 2024 US 2024/0090493 A1).

## Multimodal deterrents

Animals may acclimate to a single stimulus, and it remains unclear the underlying mechanism responsible for the negative phonotaxis in the carp (i.e., discomfort, annoyance, and pain) and habituation to the stimulus and/or carp’s motivation state (i.e., hunger, reproduction) could compel fish to pass through the barrier. It was hypothesized that intermittent pairing of the sound stimulus with a noxious stimulus (i.e., electricity, carbon dioxide) would condition the fish and produce greater efficacy with subsequent acoustic only application. Both common (Sloan et al. 2013) and grass carp (Willis et al. 2002) have been conditioned to associate sound with feeding indicating the carp can be conditioned. All fish species display aversion to high levels of dissolved carbon dioxide in the water and will swim away from these concentrations when possible and bigheaded carp avoid high concentrations in the water column (Cupp et al. 2021; Raboin et al. 2023). Carbon dioxide can induce disequilibrium and eventually death but is not species specific. However, its application in locks would be short-term and downstream currents could propel all fish (invasive and natives) away from and/or dilute the plume to minimize detrimental effects to native species. To reduce carbon dioxide applications to the environment, it is envisioned that the conditioning period (sound and CO_2_) would be intermittent and acoustic only deterrents would be used between reinforcement periods, with the conditioning enhancing the effectiveness of the acoustic deterrent.

**Fig. 1:**
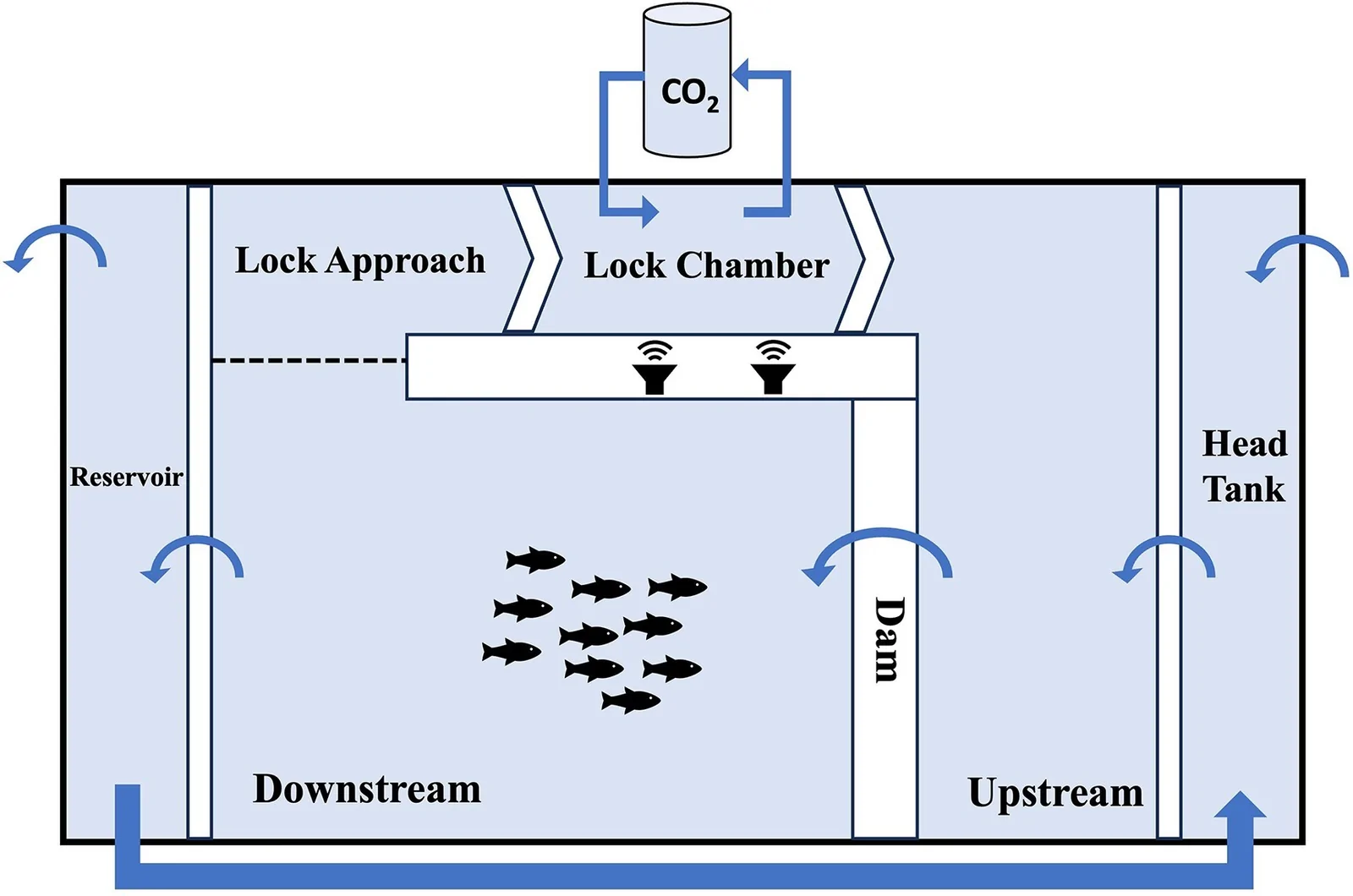
Sound and CO_2_ trials were conducted in a 10,000 L (420 cm width × 200 cm length) recirculating model lock and dam tank. The tank was divided into four zones: The upstream pool (59 cm width × 200 cm length × 52 cm water depth), lock chamber (87 cm × 40 cm × 41 cm), lock approach (73 cm × 40 cm × 42 cm), and downstream pool (256 cm × 160 cm × 42 cm). The lock chamber contained two wicket-style mechanical gates, two underwater speakers, and a CO_2_ injection system. Carp movement between the upstream and downstream pools was only possible via the lock. Water was recirculated continuously from the downstream outlet to the upstream intake with a flow rate of ~187 L/min. Water spilled over the dam wall when the gate(s) were closed, and water flowed through the lock chamber when both gates were open. Arrows indicate water flow direction. Underwater speakers are embedded in the lock chamber wall. Modified from Frett et al. (2025).

**Fig. 2:**
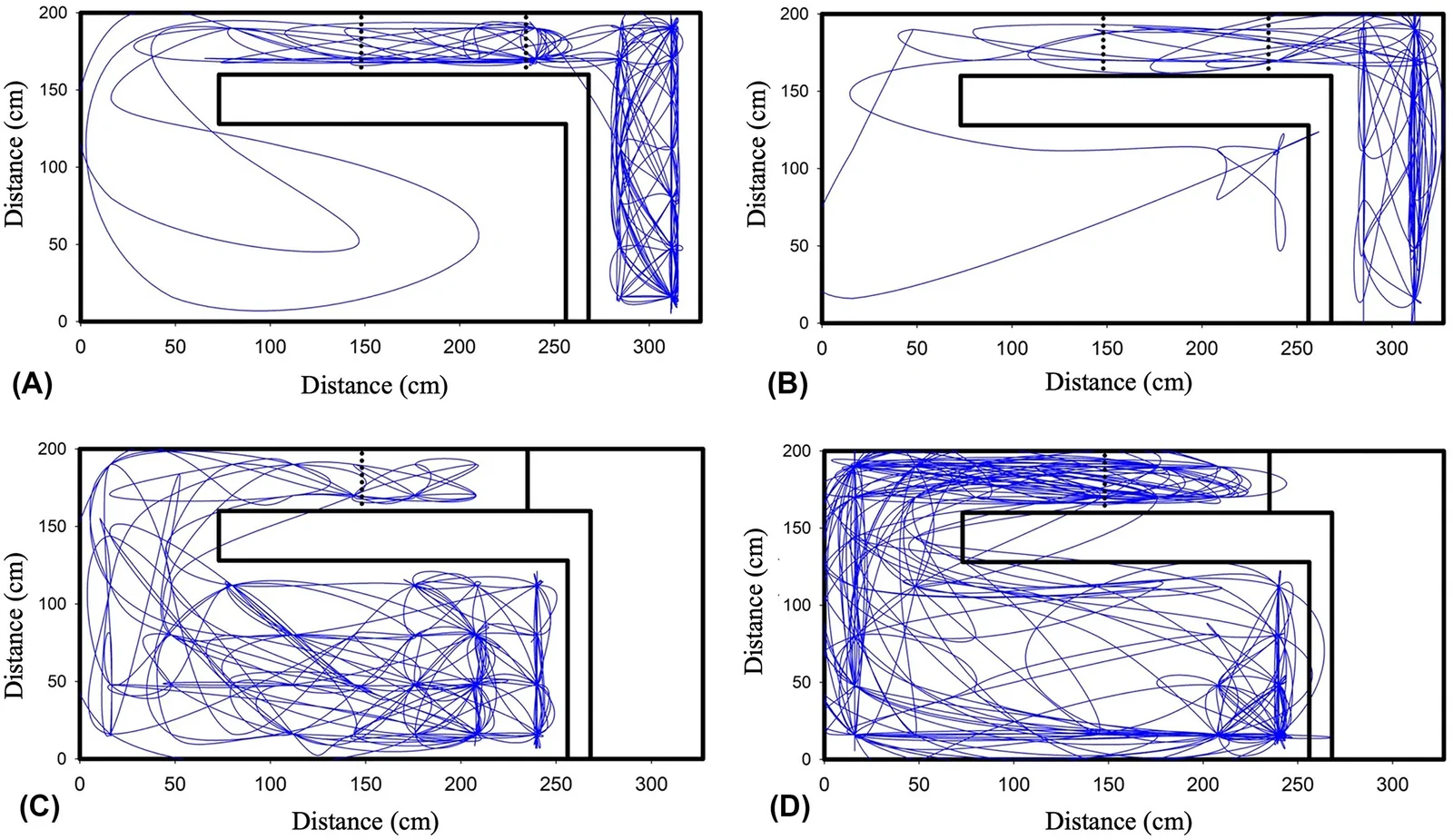
Representative fish tracking map during with lock gate open (A, B) and no acoustic deterrent and lock gate closed with acoustic deterrent on (C, D). The approximate center location of the school was recorded every 5 (C, D) or 30 s (A, B) for silver (A, C) and bighead carp (B, D) and the points were connected with spline lines. Solid (closed) and dotted (open) lines in the lock chamber indicate gate position. Figure modified from Frett et al (2025).

A preliminary study in two choice tanks indicated that carp could be conditioned to associate carbon dioxide with sound (0.06–5 kHz, ~150 dB re. 1 µPa) and show continuous aversion to sound only deterrents following conditioning (Culotta et al. 2025). Subsequent studies in a 10,000 l tank with a model lock and dam conditioned bigheaded carp with carbon dioxide and sound six times over a two-day period in the lock chamber (Fig. 1). The sound consisted of an underwater boat motor recording that had its greatest energy between 2 and 4 kHz to minimize potential effects on native nonostariophysan fishes. Sound pressure levels were 150 dB re. 1 µPa in the chamber with the sound gradient gradually decreasing to ambient levels (90 dB) by the end of the lock approach. The lock openings mimicked navigational operations with the downstream gate opened for 30 min openings four times daily during which acoustic only deterrent was used (Putland and Mensinger 2019b). After 30 min, the downstream gate was closed, the upstream gate opened, and fish in the lock could transit into the upstream pool. The study was the first to show 100% deterrence of bigheaded carp as no fish from any of the eight schools (*N* = 10 fish per school) of bighead or silver carp entered the upstream pool during the 1 week post condition (28 lock openings per week, 224 total openings) and did not show habitation to the sound as they continue to avoid the chamber throughout the study (Frett et al. 2025) (Fig. 2). However, these fish were fed daily in the downstream pool and there is concern that the fish’s physiological state (i.e., hunger and reproduction) could drive fish past the acoustic barrier. Subsequent studies restricted food delivery to the upstream pool and therefore fish were required to transit the lock to access food during the entire challenge. However, even with food-motivated fishes, there were zero upstream crossings (Frett et al. 2026)

While the Frett et al. (2025) study was the first to show 100% deterrence, laboratory studies cannot replicate the real world. Six combined exposures over a two-day period were sufficient to achieve 100% deterrence, and although bigheaded carp congregate near dams, not all will be in or near the lock chamber during the multimodal conditioning. The conditioning may extend to include fish in the lock approach as sound will project and the CO_2_ plume will drift downstream albeit at lower sound pressure levels or CO_2_ concentrations. However, portions of the resident populations or new immigrants may avoid the conditioning periods.

The combined expense, logistics, and potential environmental concerns of injecting carbon dioxide into waterways dictate intermittent use of the conditioning protocol (Frett et al. 2025), however, whether the acoustic deterrent alone will be sufficient to deter naïve fish remains to be determined. Bigheaded carp display strong, heterospecific schooling, and often aggregate downstream of locks and dams due to infrequent passage opportunities (Wilcox et al. 2004; Ghosal et al. 2016) and it is hypothesized that conditioned fish could influence naïve fish. Experiments using mixed schools of three conditioned and seven naïve fish showed only a single naïve fish in one school (*n* = 8 schools) passing through the acoustic only deterrent over 224 lock opening indicating that conditioned fish can influence the behavior of naïve fish (Vannurden and Mensinger unpublished). Native fishes including bigmouth buffalo, channel catfish fathead minnow, white sucker, lake sturgeon, and largemouth bass that were exposed to the conditioning protocol passed through the lock chamber to reach the upper pool during the acoustic only deterrent indicating that the multimodal deterrent has minimal effect on these native species.

## Translational science

Past and current basic research in animal sensory physiology can form the translational foundation to design and implement terrestrial and aquatic acoustic deterrents to focus on target species. Auditory evoked potentials (AEP) while limited in determining absolute auditory sensitivity, provide the ability to screen a large number of species for frequency sensitive which is arguably more important in acoustic deterrents (Mensinger 2024). Questions can be answered quickly under controlled lab conditions that may take several field seasons to address. Once negative phonotaxis is established, then follow-up studies in large outdoor tanks or ponds provide the next logical step to test the deterrents. However, it is difficult to create the river hydrology in these settings. Field deployments are the next step as a host of other factors can interfere with deterrent. For deterrents to be effective, they must be louder than environmental or anthropogenic sound but below levels where sensory systems can be compromised. Waters currents, weather, or ship passage can disrupt bubble curtains and allow fish passage through deterrents. They can also add to ambient sound and increase background levels and make the deterrent less effective. Ensonifying or filling a navigation lock chamber with carbon dioxide is orders of magnitude different than creating these conditions in a model lock and dam.

## Ecological impacts

Acoustic deterrents have been promoted as environmentally less impactful than other deterrents such as high head dams or electrical barriers. However, the addition of anthropogenic sound to any aquatic habitat can be defined as noise pollution and may have unintended environmental consequences to native species. The bigheaded carp deterrents are primarily generated between 2 and 4 kHz to minimize the effects on species that do not detect sound above 1 kHz. However, in the Mississippi River, there are several ostariophysans that overlap with the carp and could be affected including bigmouth buffalo (*Ictiobus cyprinellus*), channel catfish (*Ictalurus punctatus*), and fathead minnow. These species plus the invasive carp were exposed to playback of broadband sound 142 dB re: 1 µPa (max energy 2 to kHz), with negative phototaxis ranging from strong (Bighead) to moderate (silver and grass carp) to low (common carp, bigmouth buffalo) with the other species not displaying any reaction to the sound (Murchy et al. 2022). Similar results were observed in the model lock and dam with only the bigheaded carp be deterred and native fishes passing to the upstream pool (Vannurden and Mensinger unpublished)

## Chronic acoustic exposure

It is currently hypothesized that a sound source of 170 to 180 dB will be needed to establish an effective sound gradient in the lock chamber or approach to allow the fish to turn around. These sound levels are above levels that induced acute or transient hearing loss over extended time periods in fishes and raise concern for physiological damage (Smith et al. 2004). Unlike in laboratory studies, fish can swim away from the sound and therefore, it is predicted if a sound gradient is well established, that fish will only be transiently exposed to damaging sound, before reversing course. Additionally, in the Mississippi River, most native species do not detect frequencies above 1 kHz and the 2 to 4 kHz deterrent should have minimal effect on these species. However, native ostariophysans will need to be carefully monitored to ascertain that the sound is not damaging their auditory systems or preventing natural migration.

While the main river channel, surrounding wetlands and riverbanks may be teeming with wildlife, lock chambers have never been considered wildlife refuges. The location is heavily impacted by human activity and the proposed deployments areas have a high degree of ambient sound including water movement, vessel traffic, and machinery which increases background sound above the main river channel (Putland et al. 2021). Unlike in open ocean, acoustic signals can be rapidly attenuated due to the narrow, relatively shallow channel, concrete infrastructure, and soft bottom which would prevent the sound from carrying into the main channel or downstream past the lock approach. Amphibians, aquatic reptiles, birds, and mammals (i.e., otters, beaver) are more prevalent in the main channel or along the riverbanks than in the lock chambers and approaches and have sufficient mobility to move away from or avoid areas of high sound intensity or carbon dioxide concentrations and therefore the environmental consequences should be minimal.

Carbon dioxide application to the lock chamber is an environmental concern. The gas produces transient acidic effects in the water that may provide additional challenges to nontarget species but as the lock has relatively low species diversity and the CO_2_ will be quickly diluted in the open river, the effects may be localized. The carbon dioxide for the model lock and dam studies was an industrial byproduct, and no new gas was generated for the experiments. It would be optimal that byproducts be tapped for field experiments and new gas not generated for deterrents to mitigate the environmental effects. The proposed intermittent use of carbon dioxide will also reduce potential effects and with seasonal closures due to ice, maintenance, and/or water levels the locks are often not operated year-round further reducing carbon dioxide applications. Additionally, it appears that carp migration season generally peaks in spring and early summer (Fritts et al. 2024) which would allow carbon dioxide applications to be less frequent the rest of the year.

## Future directions

The field deployments of acoustic deterrents at Barkley Dama and Lock and Dam 19 with future deployments scheduled at Brandon Roads and Lock and Dam 5 show the translation power of basic research. The current sites have extended monitoring to properly quantity to efficacy of the deterrents and the engineered sound at Lock and Dam 19 is available for examination under a pending patent. One issue is that the sites use acoustic telemetry tags and processing time precludes real-time observations and depending on the tag operating frequency, fish movements may be missed. Use of real-time forward-facing sonar that reveals how fish interact with deterrents could allow fine tuning and greater deterrence during specific seasonal and river conditions.

Greater transparency and details in acoustic signals, soundscape mapping, equipment fish tracking/position, efficacy, and potential habituation would allow better comparison of methods and techniques and allow more rapid development of new deterrents.

Finally, most studies report sound pressure levels even though all fishes also detect particle motion. Historically, hydrophones availability, expense and deployment have been simpler than placing accelerometers in the water. However, to completely understand how fish interact with acoustic stimuli, both parameters should be reported. Development of low-cost field accelerometers would allow greater comprehension of how fish interact with both sound pressure and particle motion

## Summary

Acoustics deterrents continue to show promise in reducing interactions with anthropogenic hazards and limiting upstream passage. Laboratory experiments allow fish hearing ranges to determined and controlled studies to assess fish interactions with sound. However, they are constrained by the inability to recapitulate the soundscape or hydrology of field deployments. Larger outdoor ponds/tanks provide an avenue to translate basic science into more realistic deterrent scenarios. The field has been constrained by the lack of long-term monitoring and specific details on the acoustic deterrents and the independently monitored deterrents at Barkley Dam and Lock and Dam 19 are positive developments for the field. Multimodal deterrents have the potential to be more effective than single stimulus as evidenced by the bigheaded carp studies.

## Data Availability

Data are available from the author or alternatively the multimodal deterrent data is available at Data Repository for U of M (DRUM). Persistent link for this collection https://hdl.handle.net/11299/166578.
